# A Posteromedial Portal Allows Access to the Posteromedial Knee, While a Posterolateral Portal Risks Common Fibular Nerve Injury: A Cadaveric Analysis

**DOI:** 10.1016/j.asmr.2023.100880

**Published:** 2024-01-28

**Authors:** Kelsi Greenwood, Nkhensani Mogale, Reinette Van Zyl, Natalie Keough, Erik Hohmann

**Affiliations:** a Department of Anatomy, School of Medicine, Faculty of Health Sciences, University of Pretoria, South Africa; b Department of Clinical Anatomy and Imaging, Warwick Medical School, The University of Warwick, Warwick, UK; c Burjeel Hospital for Advanced Surgery, Dubai, United Arab Emirates; d School of Medicine, Medical School, University of Pretoria, South Africa

## Abstract

**Purpose:**

To investigate the safety and accessibility of direct posterior medial and lateral portals into the knee.

**Methods:**

This study was a controlled laboratory study that comprised a sample of 95 formalin-embalmed cadaveric knees and 9 fresh-frozen knees. Cannulas were inserted into the knees, 16 mm from the vertical plane between the medial epicondyle of the femur and the medial condyle of the tibia, and 8 (females) and 14 mm (males) from the vertical plane connecting the lateral femoral epicondyle and lateral tibial condyle. Landmarks were identified in full extension, and cannula insertion was completed with the formalin-embalmed knees in full extension and the fresh-frozen knees in 90 degrees of flexion. The posterior aspects of the knees were dissected from superficial to deep to assess potential damage caused by the cannula insertion.

**Results:**

The incidence of neurovascular damage was 9.6% (n = 10): 0.96% for the medial cannula and 8.7% for the lateral cannula. The medial cannula damaged 1 small saphenous vein (SSV). The lateral cannula damaged 1 SSV, 7 common fibular nerves (CFNs), and both the CFN and lateral cutaneous sural nerve in 1 specimen. All incidences of damage occurred in formalin-embalmed knees. The posterior horns of the menisci were accessible in all specimens.

**Conclusions:**

A direct posterior portal into the knee with reference to the medial bony landmarks of the knee proved safe in 99% of the cadaveric sample and allowed access to the posterior horn of the medial meniscus. A direct posterior portal with reference to the lateral bony landmarks demonstrated a higher risk of neurovascular damage in the embalmed sample but no damage in the fresh-frozen sample. Given the severe consequences of common fibular nerve injury, recommending this approach at this stage is not advisable.

**Clinical Relevance:**

Direct posterior arthroscopy portals are understudied but may allow safe visualization of the posterior knee compartments and may also assist to manage repair of ramp lesions and posterior meniscus pathology.

Knee arthroscopy typically makes use of portals on the anterior, medial, and lateral aspects of the knee.^1^^,^^2^ However, access to the posterior compartment of the knee through these standard portals is challenging and may result in either poor visualization of the posterior structures or difficulties to address pathology.^3^, ^4^, ^5^ Anterior portal access has been reported to allow limited instrument advancement^4^^,^^5^ and gaps in visualization in the posteromedial corner of the joint capsule/knee joint.^3^

“Arthroscraping” is the risk associated with anterior portal use; the term describes the iatrogenic cartilage damage caused by the advancement of the arthroscopic device in the tight medial compartments.^6^ The posteromedial and posterolateral portals provide a potential solution to the mobility and visualization issues presented by the anterior portals. However, these portals hold higher risk for iatrogenic nerve damage to the structures that pass through the popliteal fossa.^7^^,^^8^ Inaccurate positioning of the posteromedial^7^ and posterolateral portals poses risks of damage to the saphenous nerve and the common fibular nerve, respectively.^8^

The dense field of neurovascular structures situated in the popliteal fossa forms a motive for surgeons to overlook this aspect of the knee as a means of access into the posterior compartment.^9^ However, a direct posterior approach to the knee holds the potential to eliminate the hurdles associated with the standard portals described above. A direct posterior portal would possibly reduce the complications associated with all-inside devices for the treatment for posterior horn meniscal tears.^10^ Other advantages include lowering the incidence of iatrogenic cartilage damage due to the navigation into the posterior compartment from standard anterior portals^6^ and increased visualization for meniscal ramp lesion diagnosis.^11^ Recently, Greenwood et al.^12^ have reported anatomic safe zones for direct posterior portal placement into the knee.

The purpose of this study was to investigate the safety and accessibility of direct posterior medial and lateral portals into the knee. Our hypothesis was that both portals would prove as a safe and clinically useful approach to the posterior compartment of the knee.

## Methods

This study was designed as a controlled laboratory study. Formalin-embalmed cadavers were obtained from the Department of Anatomy at the University of Pretoria, South Africa. These specimens were donated and used for the anatomy courses for medical and dental students. The embalmed cadavers are used for training and research and comply with all the requirements set out in the National Health Act 63 of 2003. Fresh-frozen samples were procured from the Global Anatomy Project LLC through Science Care, Inc. The sample comprised 95 formalin-embalmed cadaveric (mean age, 69.2 years; range, 18-99 years; males, n = 53; females, n = 42) and 9 fresh-frozen cadaveric (mean age, 66.6 years; range, 60-91 years; males, n = 5; females, n = 4) knees.

Specimens were included if they had no obvious macroscopic damage with an intact knee capsule, no signs of obvious trauma, previous surgery, other obvious pathology, or fractures. Cadavers were excluded if there were signs of previous vascular surgery, pathology of the popliteal fossa, and any obvious macroscopic visual signs of osteoarthritis with osteophyte formation and/or loss of articular cartilage. Specimens with Baker’s cysts, neurovascular damage in the popliteal fossa, or surgery to the neurovascular structures were also excluded from the sample.

The formalin-embalmed cadaveric knees were used to confirm the accuracy of the Greenwood et al.^12^ safe zones. Cannula insertion was performed in the prone position and with the knees fully extended. This approach was used to replicate the methodology described by Greenwood et al.^12^ Once these safe zones were confirmed as accurate, the safe zones were then tested in a sample of 9 fresh-frozen knees. Portal placement was performed in 90 degrees of flexion to mimic a clinical setting and provide more clinically translatable results more closely.

### Technique of Portal Insertion

**Fig 1:**
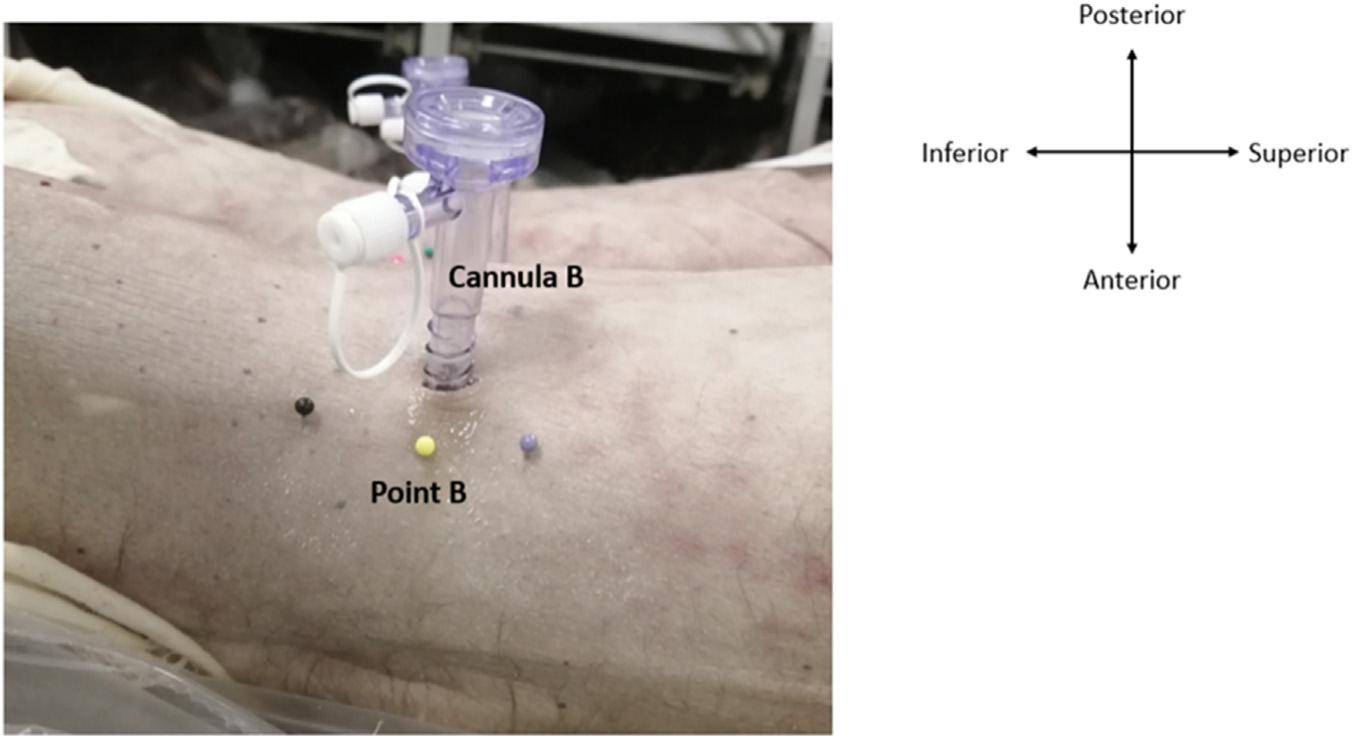
Lateral view (right knee) of point B in relation to cannula B. (Key: Black pin—lateral condyle of the tibia; yellow pin—point b; purple pin—lateral epicondyle of the femur.)

**Fig 2:**
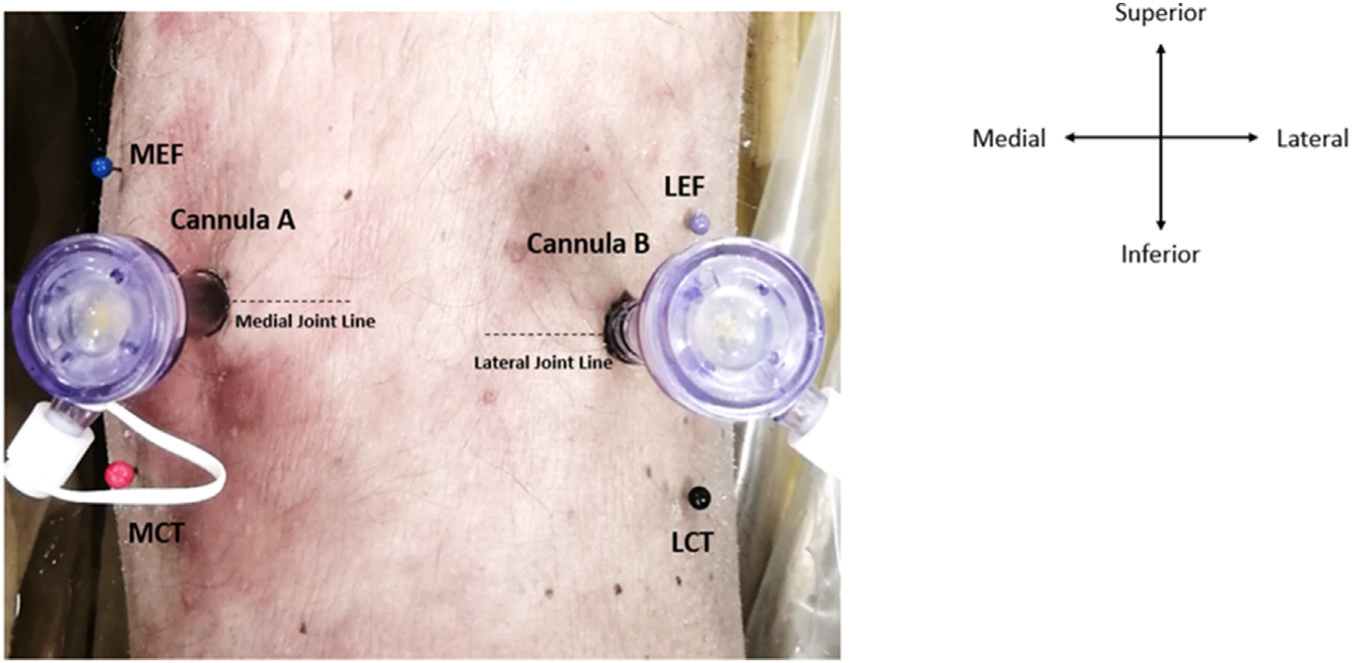
Posterior left knee with cannula A and cannula B inserted into the direct posterior portals on the medial and lateral joint lines, respectively. The following bony landmarks were pinned: medial epicondyle of the femur (MEF), medial condyle of the tibia (MCT), lateral epicondyle of the femur (LEF), and lateral condyle of the tibia (LCT).

With the cadaver in a prone position and the knees in full extension, the following landmarks were identified and marked: the medial most points of the medial epicondyle of the femur and medial condyle of the tibia and the lateral most points of the lateral epicondyle of the femur and lateral condyle of the tibia. On the medial-lateral plane between the medial femoral epicondyle and tibial condyle, the point of the medial joint line was pinned (Fig 1). Similar, the point of the lateral joint line between the lateral femoral epicondyle and tibial condyle was pinned (Fig 1). The portal positions were based on published safe zones by Greenwood et al.^12^ For the direct medial-lying posterior portal, a 6-mm diameter twist-in cannula Arthrex Twist-In (AR-6535TD) (Arthrex) was placed 16 mm lateral to point A (Figure 2). For the direct lateral portal, the cannula was placed 14 mm medial to point B in males and 8 mm medial to point B in females (Fig 2). These locations were marked, and a small vertical stab incision was performed. The cannulas were then inserted with an obturator through a small vertical incision on the proximal-distal plane of the joint line. The same technique was used for the fresh-frozen sample. However, the knee was flexed to 90 degrees. For both the embalmed cadaver and fresh-frozen specimens, the obturators were removed once the cannulas were in position.

### Outcome Measures

Once the cannulas were in position, the skin over the posterior knee was reflected and the area around the cannulas dissected to keep them in position. Dissection occurred immediately after portal placement in the formalin-embalmed cadavers, with the cadavers in the full extension prone position. The popliteal neurovascular structures were dissected from superficial to deep, and a photograph, including a scale, was taken at each level with the structures in their original positions. Both the formalin-embalmed and fresh-frozen knees remained in full extension. The neurovascular structures accounted for were the small saphenous vein, medial cutaneous sural nerve, lateral cutaneous sural nerve, tibial nerve, common fibular nerve, popliteal vein, and popliteal artery. Once all the structures were identified and photographed, the cannulas were removed. The plane of the portal placement was examined for any neurovascular structures that may have been damaged. The accessibility to the posterior horns of the menisci was also assessed.

Measurements were taken using photographs and the ImageJ software (National Institutes of Health). The distance between the lateral edge of the medial cannula and the medial edge of the small saphenous vein, medial cutaneous sural nerve, lateral cutaneous sural nerve, tibial nerve, popliteal vein, and popliteal artery was recorded. The distance between the medial edge of the lateral cannula and the lateral edge of the lateral cutaneous sural nerve and common fibular nerve was measured digitally using ImageJ software.

### Statistical Analysis

Descriptive statistics were performed and reported with 95% and 99% confidence intervals (CIs). Data normality was assessed with the Shapiro-Wilk test and visualization of the data spread. Categorical and count variables were compared using the Fisher exact test. Continuous variables were compared by means of Student *t* tests. Statistical significance was set at *P* = .01 and reported with 95% CIs. Statistical analyses were performed using R and RStudio version 4.1.2 (R Core Team, 2021).

## Results

### Damage to the Neurovascular Structures

Of the 104 cadaveric knees, there was a 9.6% rate (n = 10) of neurovascular structure damage due to the placement position of the cannula. All the incidences of damage were observed in the formalin-embalmed sample. No damage was observed in the fresh-frozen sample. Of the specimens that did not encounter damage to a neurovascular structure, all showed access to the posterior horn of the medial and lateral menisci through the portal sites. The medial-lying cannula damaged the small saphenous vein (SSV) in 1 male specimen. The lateral-lying cannula damaged the SSV in 1 male specimen, the common fibular nerve in 7 specimens (3 female, 4 male) (6.7%), and both the common fibular nerve and lateral cutaneous sural nerve in 1 male specimen. The overall incidence of damage by the medial-lying cannula was 0.96% (n = 1) compared to 8.7% (n = 9) by the lateral-lying cannula (*P* = .019; 95% CI, 0.002-0.77) (Table 1). The popliteal neurovascular bundle comprising the tibial nerve, popliteal vein, and popliteal artery was not damaged by a direct posterior cannula.

**Table 1:** Damaged Structure Count for the Medial and Lateral-Lying Cannulas

	Medial	Lateral	*P* Value	95% CI	99% CI
Damage	1	9	.019	0.002-0.77	0.0005-1.16
No damage	103	95			

NOTE. Fisher exact test.

CI, confidence interval.

### Measurements From the Medial-Lying Direct Posterior Cannula

In the formalin-embalmed sample, the tibial nerve, popliteal vein, and popliteal artery measured 25.53 ± 9.37 mm, 25.61 ± 8.7 mm, and 26.35 ± 9.48 mm lateral to the cannula, respectively. There was no significant difference between the sexes (*P* > .1) for these measurements. In the fresh-frozen sample, the tibial nerve, popliteal vein, and popliteal artery measured 54.46 ± 19.02 mm, 56.63 ± 14.95 mm, and 49.24 ± 19.4 mm lateral to the cannula, respectively (Table 2). The superficial structures—SSV and medial and lateral cutaneous sural nerves—measured distances from the cannulas as follows: SSV, 24.51 ± 10.12 mm in the formalin-embalmed knees and 51.45 ± 16.63 mm in the fresh-frozen knees. The medial cutaneous sural nerve measured 23.42 ± 8.79 mm in the formalin-embalmed and 48.87 ± 23.3 mm in the fresh-frozen cadavers. The lateral cutaneous sural nerve measurements from the medial-lying cannula were recorded as 33.99 ± 10.94 mm in formalin-embalmed cadavers and 63.49 ± 19.64 mm in the fresh-frozen cadavers (Table 2). All the measurements from the medial-lying direct posterior cannula showed a significant difference between the formalin-embalmed and fresh-frozen specimen groups (Table 2).

**Table 2:** Measurements From the Medial-Lying Direct Posterior Cannula

Cannula to	Embalmed Knees		Fresh-Frozen Knees		Difference in Means	
	Mean ± SD	99% CI of Measurement Range	Mean ± SD	99% CI of Measurement Range	*P* Value	95% CI
SSV	24.51 ± 10.12	21.79-27.24	51.45 ± 16.63	32.85-70.04	<.001	16.34-33.38
MCSN	23.42 ± 8.79	21.04-25.80	48.87 ± 23.30	22.81-74.93	<.001	10.78-35.3
LCSN	33.99 ± 10.94	30.86-37.12	63.49 ± 19.64	41.52-85.45	<.001	17.2-40.16
TN	25.53 ± 9.37	23.00-28.06	54.46 ± 19.02	33.19-75.73	<.001	16.94-36.35
PV	25.61 ± 8.70	23.27-27.96	56.63 ± 14.95	39.91-73.36	<.001	21.56-39.78
PA	26.35 ± 9.48	23.79-28.91	49.24 ± 19.40	27.54-70.94	<.001	16.17-35.26

CI, confidence interval; LCSN, lateral cutaneous sural nerve; MCSN, medial cutaneous sural nerve; PA, popliteal artery; PV, popliteal vein; SSV, small saphenous vein; TN, tibial nerve.

### Measurements From the Lateral-lying Direct Posterior Cannula

**Fig 3:**
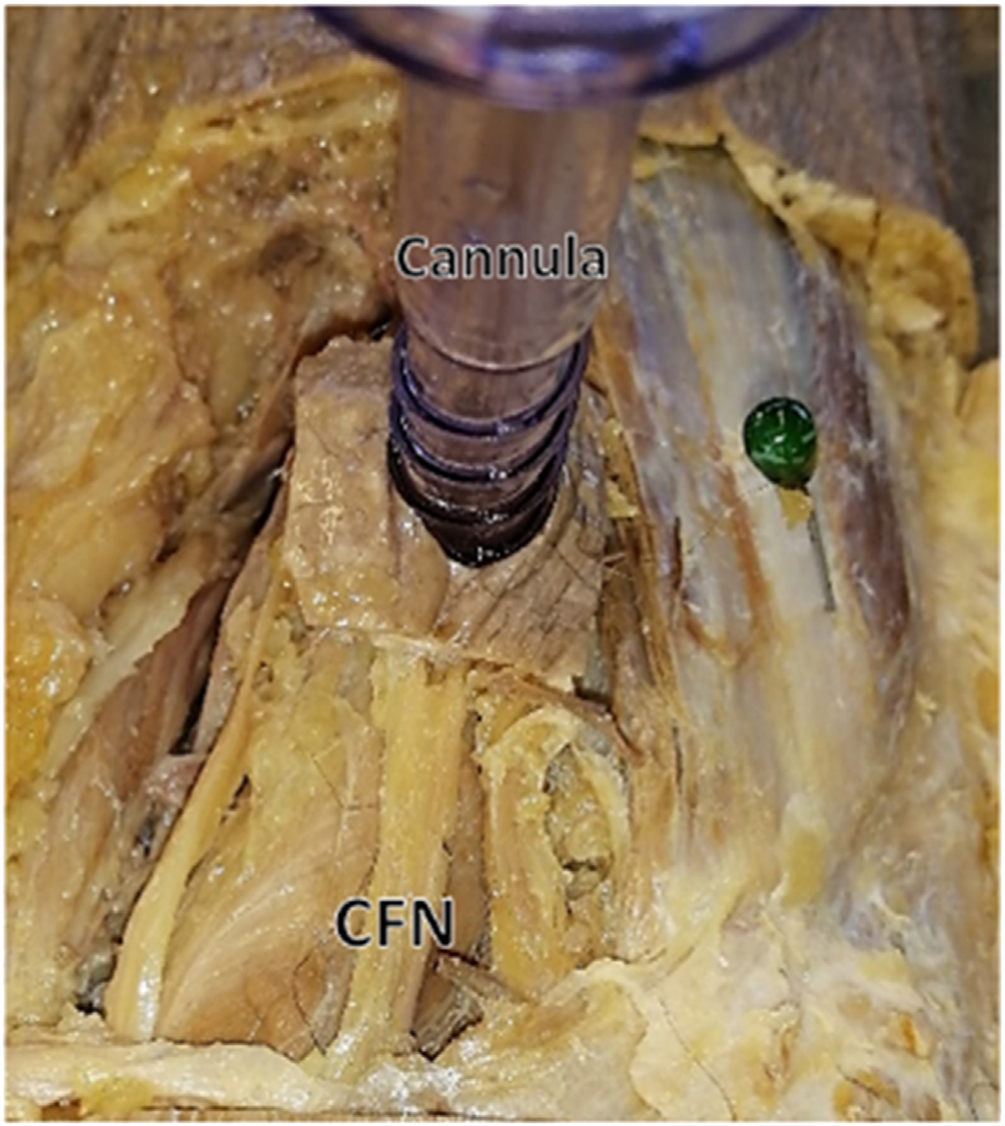
The lateral-lying direct posterior cannula demonstrating a damaged common fibular nerve (CFN) upon placement (right knee).

Of the sample, only 13.5% (n = 14) of the common fibular nerves were located lateral to the cannula with a mean distance of 8.09 ± 3.88 mm. On the other hand, the majority of the sample, 78.8% (n = 82), presented with the common fibular nerve medial to the lateral cannula with a distance of 10.22 ± 7.69 mm from the cannula. The remaining 7.7% (n = 8) were damaged on insertion of the lateral-lying cannula (Table 3; Fig 3).

**Table 3:** Measurements From the Lateral-Lying Direct Posterior Cannula

Cannula to	Embalmed Knees		Fresh-Frozen Knees		Difference in Means	
	Mean ± SD	99% CI of Measurement Range	Mean ± SD	99% CI of Measurement Range	*P* Value	95% CI
LCSN	19.11 ± 10.14	16.23-21.99	25.03 ± 11.62	12.04-38.02	.19	2.34-15.35
CFN (medial)	10.22 ± 7.69	7.98-12.47	11.75 ± 10.49	0.03-23.48	.14	1.68-8.72
CFN (lateral)	8.09 ± 3.88	4.80-11.37	N/A	N/A	N/A	N/A

CFN, common peroneal nerve; CI, confidence interval; LCSN, lateral cutaneous sural nerve; MCSN, medial cutaneous sural nerve; SSV, small saphenous vein.

The lateral cutaneous sural nerve was measured from the lateral-lying cannula and documented as 19.11 ± 10.14 mm for the formalin-embalmed cadavers and 25.03 ± 11.62 mm in the fresh-frozen knees (Table 3). The measurements from the lateral-lying direct posterior cannula did not significantly differ between the formalin-embalmed and fresh-frozen specimen groups (Table 2).

## Discussion

The results of this study demonstrated that the direct posterior and medial-lying cannulas are safe in 99% of the sample in this study. The SSV was damaged in 1 embalmed cadaver; no damage was observed in the fresh-frozen specimens. The lateral-lying direct posterior portal was safely inserted in 91% of the sample. The lateral-lying direct posterior cannula showed a significantly higher incidence of neurovascular damage at 8.7% when compared to the damage rate of the medial-lying direct posterior portal. There were 7 counts of common fibular nerve damage, 1 count of lateral cutaneous sural nerve damage, and 1 count of SSV damage.

However, the fresh-frozen cohort saw no incidence of damage and reported significantly larger distances between the medial-lying cannula and the neurovascular structures (*P* < .001). The results from the study align with the results from Greenwood et al.,^12^ which suggested a 99% success rate of safe insertion if the cannula was placed no greater than 20 mm from the medial bony landmarks.

A statistically significant difference is noted between fresh-frozen and formalin-embalmed medial measurements. For example, the popliteal artery measurements were 26.35 ± 9.48 and 49.24 ± 19.40 mm, respectively (*P* < .001; 95% CI, 16.17-35.26). The study is, however, not a comparison between the 2 tissues, but a horizontal progression of a technique analysis. The formalin-embalmed measurements showed small but safe distances between the neurovascular structures and the medial cannula. When repeating the technique in fresh-frozen knees, these distances were larger and still showed damage to neurovascular structures by the medial cannula. Inserting the portals in a 90-degree flexion position will place them further away from neurovascular structures. The significant difference in measurement magnitude could be attributed to the difference in tissue characteristics and rigidity between the 2 samples^13^ and the difference in flexion vs extension position when inserting the cannulas.

The posterior horn of the medial meniscus was accessible through the portal in all specimens. This could assist in the visualization and identification of meniscal ramp lesions, which has proved difficult through anterior viewing portals^11^ and magnetic resonance imaging.^14^ It could also minimize the high incidence (77.8%), noted by Compton et al.,^6^ of iatrogenic cartilage damage during meniscal repairs by potentially providing a means of access that avoids the cartilage entirely.

Damage to the common fibular nerve can cause foot drop, abnormal gait, and sensory loss of the anterolateral lower leg or dorsum of the foot.^15^ Due to the high incidence of neurovascular damage (8.7%), the common fibular nerve in particular (6.7%), the lateral-lying posterior portal was not a viable option in this position. There was no incidence of damage to the popliteal neurovascular bundle (tibial nerve and popliteal artery and vein) noted in this study. The structures in this bundle are of extreme clinical importance and must avoid damage at all costs.^9^ This study demonstrated the preservation of these structures when performing the direct posterior portal technique.

The superficial structures, SSV and medial and lateral cutaneous sural nerves, are highly variable in terms of their positioning within the popliteal fossa.^16^, ^17^, ^18^ This could account for the 1.9% and 0.96% incidence of damage in the SSV and lateral cutaneous sural nerve, respectively. The risk of damage to these superficial structures upon insertion of the cannulas is relatively low. The lateral cutaneous sural nerve supplies sensory innervation to a small portion of the lateral knee and proximal leg. This nerve is often harvested for reconstructive surgery with mild sensory loss, discomfort, and complications postoperatively.^19^

It should be noted that this study specifically concentrated on the knee in a singular fully extended position. Existing studies highlighted that the position of the neurovascular structure varies in flexion.^20^, ^21^, ^22^, ^23^ Kim et al.^20^ and Yoo and Chang^23^ independently reported that the popliteal artery (PA) was located more posterior in flexion. Additionally, Thi et al.^22^ reported a reduction in distance between the common fibular nerve and the fibula head with increasing knee flexion. Notably, the tibial nerve, popliteal vein, and popliteal artery demonstrated a posterior and lateral shift when comparing its position in extension and 90 degrees of flexion of the knee.^23^ This implies that the safe interval for a direct posterior medial portal, relative to the medial femoral condyle, expands with knee flexion due to the lateral displacement of these neurovascular structures. Ogilvie-Harris et al.^24^ demonstrated that both the saphenous vein and nerve as well as the common fibula nerve became more mobile and falling behind the biceps femoris but also shifted more medially. Nevertheless, these observations may not necessarily mitigate the risk of injury to the lateral neurovascular structures, and as of now, advocating for direct posterior portal placement is not advisable.

### Limitations

This study is not without limitations. The stiffness of embalmed tissue^13^ and the potential anatomic changes as a result of tissue preservation^25^ are limitations in this study. However, Kennel et al.^26^ advised that embalming does not cause changes in the anatomy. These uncertainties were counteracted by including a sample of fresh-frozen cadaveric knees. Another limitation is the different positioning used between the 2 types of cadaveric knees for cannula insertion. Formalin-embalmed cadavers were restricted to a full extension position due to the rigidity of the tissue, but this positioning is not plausible in a clinical setting. Therefore, the fresh-frozen knees were placed in 90 degrees of flexion for cannula insertion to better replicate a clinical setting and present results that are more clinically relevant. Finally, a notable limitation in this study pertains to the omission of measurements obtained in flexion.

## Conclusions

A direct posterior portal into the knee with reference to the medial bony landmarks of the knee proved safe in 99% of the cadaveric sample and allowed access to the posterior horn of the medial meniscus. A direct posterior portal with reference to the lateral bony landmarks demonstrated a higher risk of neurovascular damage in the embalmed sample but no damage in the fresh-frozen sample. Given the severe consequences of common fibular nerve injury, recommending this approach at this stage is not advisable.

## Supplementary Data

ICMJE author disclosure forms
